# Relationships between method used for bedding processing and presence of mastitis and nonmastitis pathogens in ready-to-use recycled manure solids bedding on Midwest dairy farms

**DOI:** 10.3168/jdsc.2025-0754

**Published:** 2025-05-12

**Authors:** F. Peña-Mosca, S. Godden, E. Royster, D. Albrecht, S.J. Wells, B.A.C. Crooker, N. Aulik

**Affiliations:** 1 Department of Veterinary Population Medicine, University of Minnesota, Saint Paul, MN 55108; 2 Department of Public and Ecosystem Health, Cornell University, Ithaca, NY 14853; 3 Department of Animal Science, University of Minnesota, Saint Paul, MN 55108; 4 Wisconsin Veterinary Diagnostic Laboratory, University of Wisconsin–Madison, Madison, WI 53706

## Abstract

Field studies have examined how processing methods affect mastitis pathogen levels in ready-to-use (RTU) recycled manure solids (RMS), but few have assessed their impact on nonmastitis pathogens. This cross-sectional study investigated associations between RMS processing methods and (1) mastitis pathogen levels and (2) the presence of *Mycobacterium avium* subspecies *paratuberculosis* (MAP), *Salmonella* (SAL), and *Campylobacter jejuni* (CAMP) in RMS from Midwest dairy herds. Twenty-seven dairies in Minnesota and Wisconsin were recruited to represent various RMS processing methods: raw or green solids (GRN; n = 6), drum composters (COM; n = 3), anaerobic digesters (DIG; n = 9), digesters with hot air dryers (DIG+DRY; n = 6), digesters with infrared dryers (DIG-IR; n = 1), and hot air dryers (DRY; n = 2). Farms were visited once in summer 2021 to collect slurry and postprocessed RMS samples before and after each processing step. Samples were tested for MAP (culture and PCR confirmation), CAMP (culture), and SAL (culture). Ready-to-use RMS samples also underwent aerobic culture to determine counts of coliforms, *Klebsiella* spp., *Streptococcus* spp., *Streptococcus* spp. and *Streptococcus*-like organisms (SSLO), and *Staphylococcus* spp. (cfu/cm^3^, wet basis). For analysis, dairies were grouped into 4 system types: GRN (n = 6), DIG-only (n = 9), secondary processing only (SEC; DRY or COM; n = 5), or DIG combined with SEC (DIG+SEC; n = 7). Linear regression assessed associations between processing type and mastitis pathogen counts, and logistic regression evaluated MAP and SAL presence before and after processing. No CAMP was detected. Prevalence of MAP and SAL in raw slurry was high (MAP: 68% [17/25]; SAL: 80% [21/25]). Compared with GRN, DIG-only and SEC-only systems were associated with lower mastitis pathogen counts and reduction of MAP and SAL presence, though these pathogens were still identified in RTU RMS samples. The DIG+SEC systems showed the greatest reduction in mastitis pathogen counts, and MAP and SAL were not detected in RTU RMS. Our results suggest that combining DIG with a secondary processing method (e.g., COM, DRY, or IR) most effectively reduces mastitis and nonmastitis pathogens in RMS bedding.

## Highlights

• Anaerobic digestion only reduced pathogens, but they were still detected in bedding.
• Combining digestion and secondary processing resulted in lower mastitis pathogen counts.
• Combining digestion and secondary processing resulted in no detection of *Salmonella* or *M. avium* ssp. *paratuberculosis*.

## Graphical Abstract

**Figure:**
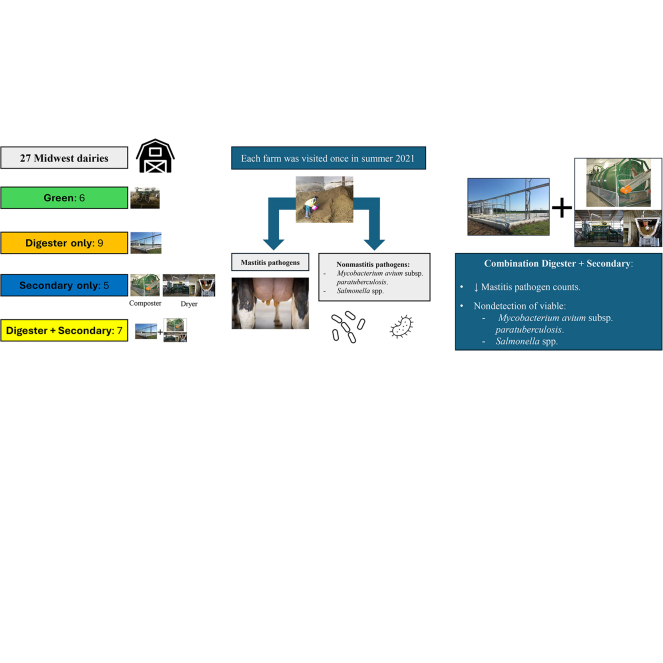
**Summary:** This study investigated associations between recycled manure solids (RMS) processing systems, used alone or in combination, on the presence of important pathogens affecting dairy cattle health. Our results showed that when used as a standalone processing technique, anaerobic digestion or secondary processing methods (e.g., composting or drying) reduced the counts of mastitis pathogens. These methods also reduced the risk of *Mycobacterium avium* ssp. *paratuberculosis* and *Salmonella* spp., but these pathogens were still detected in ready-to-use recycled RMS samples. However, combining anaerobic digestion with a secondary processing method led to the greatest reduction in mastitis pathogen counts and nondetection of *M. avium* ssp. *paratuberculosis* and *Salmonella* spp. in RMS samples. Further research is needed to confirm these findings, assess cost benefit, and examine how different processing system combinations may influence the presence of other pathogens significant to animal health.

Recycled manure solids (**RMS**) are increasingly used as bedding for dairy cows in the Midwest. Although this bedding offers an available alternative to conventional bedding materials, prior research shows that farms that use green (i.e., raw; **GRN**) RMS have higher bedding bacterial counts, and impaired udder health compared with farms using other bedding materials (Patel et al., 2019). Another concern is that use of RMS bedding could recirculate other important pathogens (some of which are zoonotic) that are shed in manure, such as *Salmonella* spp. (**SAL**), *Mycobacterium avium* ssp. *paratuberculosis* (**MAP**), and *Campylobacter jejuni* (**CAMP**; Leach et al., 2015; Nag et al., 2019). The high risk for presence of MAP or SAL in GRN RMS could lead to early exposure and infection risk, particularly for dairies using RMS bedding for youngstock (Nielsen et al., 2012; Mortier et al., 2015). Furthermore, recycling RMS potentially containing MAP and SAL could pose a biosecurity risk for “community” or “hub-and-spoke” anaerobic digesters (**DIG**), where slurry from multiple farms is comingled for digestion at a central location. Distributing the comingled finished product to cooperating farms in turn increases their risk for exposure (Thompson et al., 2013; Nag et al., 2019; US EPA, 2021) and violates the basic biosecurity principle of bioexclusion (Mee et al., 2012). Additionally, farms with extra bedding may sell it to others, raising further biosecurity concerns.

Field-based (on-farm) studies on RMS processing methods and their impact on pathogen reduction are limited (Burch et al., 2018; Mazzone et al., 2018; Donat et al., 2019). Evidence suggests that DIG can reduce some, but not all, mastitis pathogen counts and might not sufficiently lower pathogen loads to mitigate mastitis risks (Burch et al., 2018; Tran et al., 2021; Godden et al., 2023). Midwest dairy farms relying on mesophilic DIG decreased coliforms and *Klebsiella* spp. in ready-to-use (**RTU**) solids but not *Streptococcus* spp. and *Streptococcus*-like organisms (**SSLO**) compared with dairies that used GRN RTU solids (Godden et al., 2023).

Field studies have also evaluated secondary (following [or secondary to] the liquid-solid separation step; **SEC**) processing methods such as composters (**COM**) and hot air dryers (**DRY**), but studies of commercial infrared dryers (**IR**) systems are lacking (Husfeldt et al., 2012; Fournel et al., 2019; Godden et al., 2023). Our previous study found lower bedding bacterial count in RTU RMS and improved udder health in herds using COM or DRY compared with GRN. However, it included few COM farms and did not assess RMS system combinations or other health-related microorganisms (Godden et al., 2023). The objective of this study was to investigate the relationship between different types of RMS processing systems, used alone or in combination, and (1) the levels of mastitis pathogens and (2) the presence of MAP, CAMP, and SAL in RTU RMS on Midwest dairies.

A convenience sample of 27 dairies in Minnesota (n = 12) and Wisconsin (n = 15) was recruited to achieve a sample of different processing methods: GRN (n = 6), DIG (n = 9), COM (n = 3), DRY (n = 2), DIG-DRY (n = 6), and DIG-IR (n = 1). Dairies using DIG operated plug-flow systems with temperatures within the mesophilic range (∼37°C [98.6°F]). Farms were visited once in summer 2021, and slurry and solids samples were collected before and after each processing step (e.g., raw or post-digested slurry, post-pressed solids, and post-COM, post-DRY, or post-IR RTU solids).

Sampling was conducted by a trained technician wearing clean disposable gloves. Slurry was collected with a sanitized metal ladle from containers before entering the digester or screw press and placed in duplicate 50-mL sterile tubes, kept on ice, and frozen at −20°C within 8 h. For post-pressed solids, 15 random grab samples of RMS were mixed in a clean bucket and transferred into duplicate 1-quart resealable plastic bags. For RTU, grab samples were taken from the top 5 cm of the RMS pile at 15 random locations, mixed, and placed in duplicate resealable plastic bags, sealed, and put on ice before freezing at −20°C within 8 h. All samples were transported on ice to the University of Minnesota College of Veterinary Medicine, with one duplicate sent to the Laboratory for Udder Health (St. Paul, MN) and the other to the University of Wisconsin–Madison Veterinary Diagnostic Laboratory (Madison, WI).

At the University of Minnesota Laboratory for Udder Health, frozen RTU bedding samples were thawed to room temperature and processed using standard aerobic culture methods (Godden et al., 2023; Peña Mosca et al., 2024). Briefly, 50 cm^3^ of bedding was mixed with 250 mL of sterile water in a Whirl-Pak bag (Nasco), incubated for 10 min, and serially diluted. Dilutions were plated on MacConkey agar and colistin nalidixic acid agar for gram-negative and gram-positive bacteria, respectively, and incubated at 37°C overnight. Lactose fermenters on MacConkey agar were counted as coliforms, nonfermenters as noncoliform gram-negatives, and *Klebsiella* spp. were identified by MALDI-TOF MS and reported as a percentage of coliforms. On colistin naladixic acid plates, bacteria were classified as *Staphylococcus* spp., SSLO, or *Bacillus* spp. Colony counts were reported as cfu/cm^3^ of wet bedding, with a detection limit of 25 cfu/cm^3^.

Frozen slurry and solids samples were tested for MAP, SAL, and CAMP at the University of Wisconsin Veterinary Diagnostic Laboratory (Madison, WI). For SAL culture, samples were pre-enriched in buffered peptone water (1:10) and incubated at 36 ± 2°C for 18 to 24 h. Aliquots were transferred to tetrathionate broth with iodine (1:10), selenite F broth (1:10), and Rappaport-Vassiliadis R10 broth (1:100) and incubated for another 18 to 24 h. Broths were then plated on brilliant green with novobiocin (**BGN**) and xylose-lysine tergitol 4 (**XLT-4**) agars, incubated at 36°C for 18 to 24 h, and examined for SAL-indicative colonies (pink on BGN, black on XLT-4). Identification was confirmed by MALDI-TOF MS using a score threshold of 2.3 to 3.0. At least one colony type per plate was serogrouped and serotyped via the White–Kauffmann–Le Minor scheme (Grimont and Weill, 2007). For CAMP detection, samples were plated on CAMP CVA agar (Hardy Diagnostics) and incubated in a microaerophilic environment (AnaeroPack System; Mitsubishi Gas Chemical America Inc.) at 35 ± 2°C for 48 h. For MAP, 2-g RMS samples were mixed with 35 mL sterile water, vortexed, and incubated for 30 min. The supernatant (5 mL) was transferred to brain heart infusion broth with hexadecylpyridinium chloride (9 mg/mL) and incubated at 36 ± 2°C for 18 to 24 h. After centrifugation 3,000 × *g* for 20 min at room temperature, the pellet was resuspended in brain heart infusion with vancomycin (100 µg/mL), nalidixic acid (10 µg/mL), and amphotericin B (50 µg/mL) and incubated again at 36 ± 2°C for 18 to 24 h. Samples were then cultured in prepared liquid media bottles using the VersaTREK system (TREK Diagnostics) for 42 to 56 d alongside a positive control (Wells et al., 2003). We extracted DNA with the MagMAX Total Nucleic Acid kit (ThermoFisher), and real-time PCR targeted IS900, MAP2765c (251), and MAP0865 (F57) genes (Imirzalioglu et al., 2011). A positive result required 2 of 3 targets with a cycle threshold (**CT**) below 40.

All data analysis was performed in R (v4.3.2; https://www.r-project.org/; version 4.3.2), with code and output available online (https://fepenamosca.github.io/rms_bedding_midwest/). One farm using GRN solids had a missing slurry sample, and its RTU sample was therefore excluded from MAP and SAL presence analysis (before vs. after each step) but included for mastitis pathogen levels in RTU bedding. In addition, one farm using DIG+DRY solids had a missing pressed solids sample and was not included in the pre- versus post-screw press comparison, but all other samples from this farm were included in the analysis. Descriptive herd characteristics (mean ± SE) were reported and compared across processing systems using linear regression. Associations between processing system (explanatory variable) and log_10_ pathogen counts in RTU bedding were analyzed using separate linear models for coliforms, *Klebsiella* spp., SSLO, and *Staphylococcus* spp. (outcome variables). Normality was assessed via residual plots and quantile-quantile plots. The prevalence of CAMP, MAP, and SAL was reported at baseline (raw slurry), in RTU samples, and at each processing step. Logistic regression estimated the relative risk (**RR**) of a positive CAMP, MAP, or SAL test before (referent) and after each step, using the function odds_ratio_to_risk_ratio (Grant, 2014). Potential confounders offered to the models included the years using the RMS bedding system and storage duration before RTU solids collection. Confounders were selected based on their impact on the estimates, aiming to retain those that changed the estimates by more than 10%. However, none were ultimately retained in the models. Herd size was excluded due to complete confounding with processing system. Multiple comparisons were adjusted using Tukey's method.

The 27 dairies were classified into 4 processing system categories: GRN (n = 6), DIG-only (n = 9), SEC-only (n = 5), and DIG+SEC (n = 7). Processing system was associated with herd size (*P* < 0.001). Herd size was similar for GRN (mean ± SE; 888 ± 743 cows) and SEC-only (1,520 ± 814 cows, *P* = 0.94), but smaller in GRN than DIG-only (4,220 ± 607, *P* = 0.01) or DIG+SEC (3,182 ± 688, *P* = 0.13). Bulk tank SCC (×10^3^ cells/mL) was higher on dairies using GRN (399 ± 34) compared with other processing systems (DIG-only: 137 ± 25, SEC-only: 175 ± 34, DIG+SEC: 133 ± 29, *P* < 0.001). Cows' mean yearly milk production (×1,000 kg/yr; GRN: 12.8 ± 0.7, DIG-only: 12.2 ± 0.6, SEC: 13.3 ± 0.7, SEC+DIG: 12.6 ± 0.6) and time using the current RMS processing system (years; GRN: 6.8 ± 1.6, DIG-only: 6.6 ± 1.3, SEC: 6.6 ± 1.8, SEC+DIG: 7.3 ± 1.5) did not differ among systems (*P* = 0.68 and *P* = 0.99, respectively). Ready-to-use bedding samples were on average 1.4 d old at collection, with no differences among systems (days; GRN: 1.0 ± 0.9, DIG-only: 2.4 ± 0.7, SEC: 1.0 ± 1.0, SEC+DIG: 0.9 ± 0.8; *P* = 0.48).

Processing system category was associated with coliform, *Klebsiella*, and SSLO counts (*P* < 0.05), but not with *Staph* counts in RTU solids (*P* = 0.39; Table 1). Compared with GRN solids (adjusted mean ± SE [log_10_; cfu/cm^3^]: 4.53 ± 0.72), coliform counts were numerically lower in RTU solids from farms using DIG-only (3.29 ± 0.59, *P* = 0.55) and SEC-only (3.04 ± 0.79, *P* = 0.51) and significantly lower in farms using DIG+SEC (1.53 ± 0.67, *P* = 0.03). Similarly, *Klebsiella* spp. counts were lower in farms using DIG-only (0.52 ± 0.33, *P* = 0.05), SEC-only (0.00 ± 0.44, *P* = 0.02), and DIG+SEC (0.00 ± 0.37, *P* = 0.008) compared with farms using GRN solids (1.93 ± 0.40).

**Table 1:** Estimated marginal means from linear regression models investigating the association between processing system and bacterial count (BBC; log_10_ cfu/cm^3^; mean ± SE) in ready-to-use recycled manure solids used for bedding^1^

BBC (log_10_ cfu/cm^3^)	GRN (n = 6)	DIG-only (n = 9)	SEC-only (n = 5)	DIG+SEC (n = 7)	*P*-value^2^
Coliforms	4.53 ± 0.72^a^	3.29 ± 0.59^ab^	3.04 ± 0.79^ab^	1.53 ± 0.67^b^	0.04
*Klebsiella*	1.93 ± 0.40^a^	0.52 ± 0.33^ab^	0.00 ± 0.44^b^	0.00 ± 0.37^b^	0.006
SSLO	5.61 ± 0.54^a^	4.99 ± 0.44^a^	4.08 ± 0.59^ab^	2.38 ± 0.50^b^	<0.001
*Staphylococcus*	0.00 ± 0.28	0.19 ± 0.23	0.64 ± 0.30	0.00 ± 0.26	0.39

a,b Different superscripts within a row indicate means differ (*P* ≤ 0.05). *P*-values were adjusted for multiple comparisons using Tukey's adjustment.

1 SSLO = *Streptococcus* spp. and *Streptococcus*-like organisms; GRN = green; DIG = digested; SEC = secondary systems (included mechanical drum composters and dryers [hot air and infrared]).

2 Type III *P*-values evaluating the overall association between processing system and BBC.

These findings are consistent with our previous study, in which counts of these bacteria were lower in DIG, COM, or DRY than in GRN bedding (Godden et al., 2023). Other investigations have demonstrated similar effectiveness of COM (Burch et al., 2018; Fournel et al., 2019) and DIG (Husfeldt et al., 2012). Compared with GRN-only (5.61 ± 0.54), SSLO counts were lower in RTU solids from farms using DIG+SEC (2.38 ± 0.50; *P* = 0.001) or DIG-only (4.99 ± 0.44; *P* = 0.003) and only numerically lower for farms using SEC-only solids (4.08 ± 0.59; *P* = 0.16). Counts of *Staph* were near zero in RTU solids across all systems and no relationship with the RMS processing system was detected. The limited ability of DIG to reduce counts of SSLO agrees with several previous studies (Burch et al., 2018; Tran et al., 2021; Godden et al., 2023) although one observational study did report lower SSLO counts in DIG RTU RMS versus GRN samples (Husfeldt et al., 2012). These findings suggest that DIG alone may be less effective than SEC-only systems at reducing mastitis-associated bacteria in RTU RMS. Farms already using DIG may want to consider adopting a SEC system (COM, DRY or IR) because a combination of systems (DIG+SEC) achieved the greatest reduction in mastitis causing pathogens, which has been linked to a lower prevalence of subclinical mastitis (Patel et al., 2019; Godden et al., 2023).

An interesting finding was the complete absence of detectable CAMP in both slurry or solid samples, regardless of the processing system used on the farm. Although prior studies have shown a varying prevalence of CAMP among dairy farms (Knipper et al., 2022), environmental stressors are known to affect the cultivability of this bacterium (Jackson et al., 2009).

The prevalence of MAP was high in raw slurry (68%; 17/25, Figure 1A). In RTU RMS samples, MAP was detected in GRN (40%; 2/5) and SEC-only (20%; 1/5) but not in DIG-only (0%; 0/9) or DIG+DRY (0%; 0/7; Figure 1B). Regression analyses were used to estimate RR (95% CI) to compare the likelihood of detecting a pathogen in the following processing step relative to the preceding step. An RR <1.0 indicates a reduction in pathogen detection, suggesting control during that step. Although some individual steps reduced the risk, no single processing step was associated with nondetection of MAP (Figure 2A, 2C, 2E) or SAL (Figure 2B, 2D, 2F). For pre- versus post-DIG slurry, MAP RR = 0.20 [0.04, 0.74] (*P* = 0.007) and SAL RR = 0.10 [0.01, 0.62] (*P* = 0.005). For pre-pressed slurry versus post-pressed solids, MAP RR = 0.45 [0.15, 1.06] (*P* = 0.08) and SAL RR = 1.20 [0.58, 1.85] (*P* = 0.57). For pre-SEC versus post-SEC (DRY, COM, or IR), MAP RR = 0.50 [0.04, 3.10] (*P* = 0.54) and SAL RR = 0.17 [0.02, 0.95] (*P* = 0.04). The mean (±SD) CT values among samples with detected MAP were 32.4 ± 0.5 for MAP-positive raw slurry, 30.3 ± 2.5 for post-pressed solids, 37.7 for the single post-SEC MAP-positive sample, and 34.5 ± 0.8 for post-digested slurry. The prevalence of SAL was high in raw slurry (84.0%; 21/25, Figure 1C), with serotype Cerro, serogroup K, being most prevalent (GRN: 60.0% [3/5], DIG-only: 33.3% [3/9], SEC-only: 60.0% [3/5], DIG+SEC: 14.3% [1/7]). The second most prevalent serotype was Montevideo, serogroup C1 (GRN: 20.0% [1/5], DIG-only: 44.4% [4/9], SEC-only: 20.0% [1/5], DIG+SEC: 28.6% [2/7]). Other serotypes included Agona, serogroup B (SEC-only: 20.0% [1/5]); Typhimurium, serogroup B (DIG+SEC: 14.3% [1/7]); and nonmotile, serogroup E4 (GRN: 20.0% [1/5]). In RTU RMS samples, SAL prevalence was moderate in farms using GRN (60.0% [3/5]), DIG-only (33.3% [3/9]), or SEC-only (20.0% [1/5]) and absent in DIG+SEC farms (0% [0/7]). The SAL-positive RTU RMS samples contained serotype Cerro, serogroup K (GRN, DIG-only), or serotype Agona, serogroup B (SEC-only). The high prevalence of MAP and SAL in raw slurry and GRN RTU RMS samples aligns with an earlier USDA National Animal Health Monitoring System Dairy 2007 study that used pooled fecal samples, and found herd-level MAP and SAL prevalence of 70.4% and 49.4%, respectively (Lombard et al., 2012, 2013). *Salmonella* Cerro was the most prevalent serotype in that study, consistent with our findings (Lombard et al., 2012).

**Figure 1:**
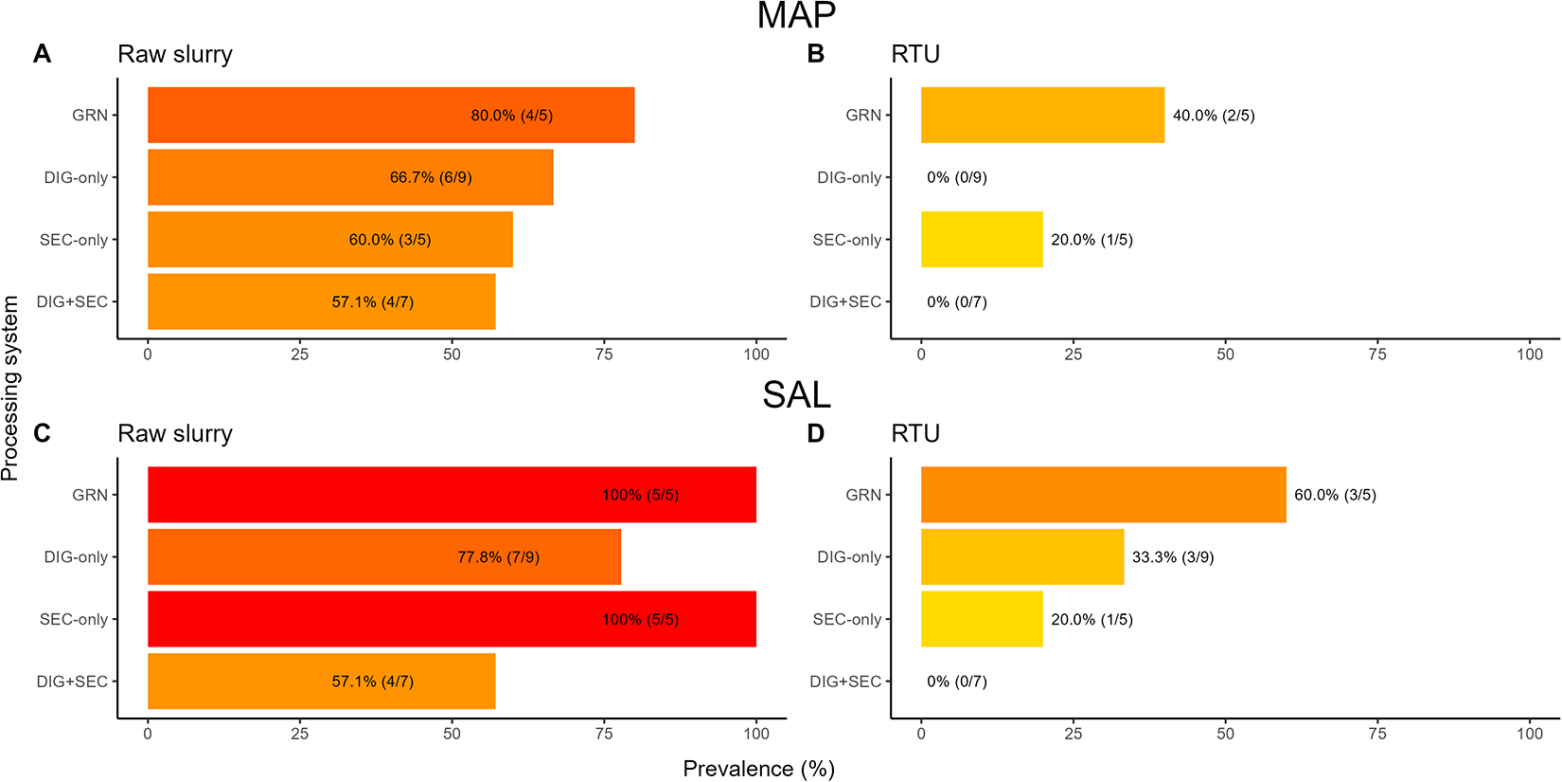
Prevalence of *Mycobacterium avium* subspecies *paratuberculosis* (MAP; panels A and B) and *Salmonella* (SAL; panels C and D) in raw slurry and ready-to-use (RTU) samples by processing system. GRN = green; DIG = digested; SEC = secondary processing systems including mechanical drum composters and driers (hot air or infrared). Bars are color-coded from yellow to red with a shift toward red indicating higher prevalence.

**Figure 2:**
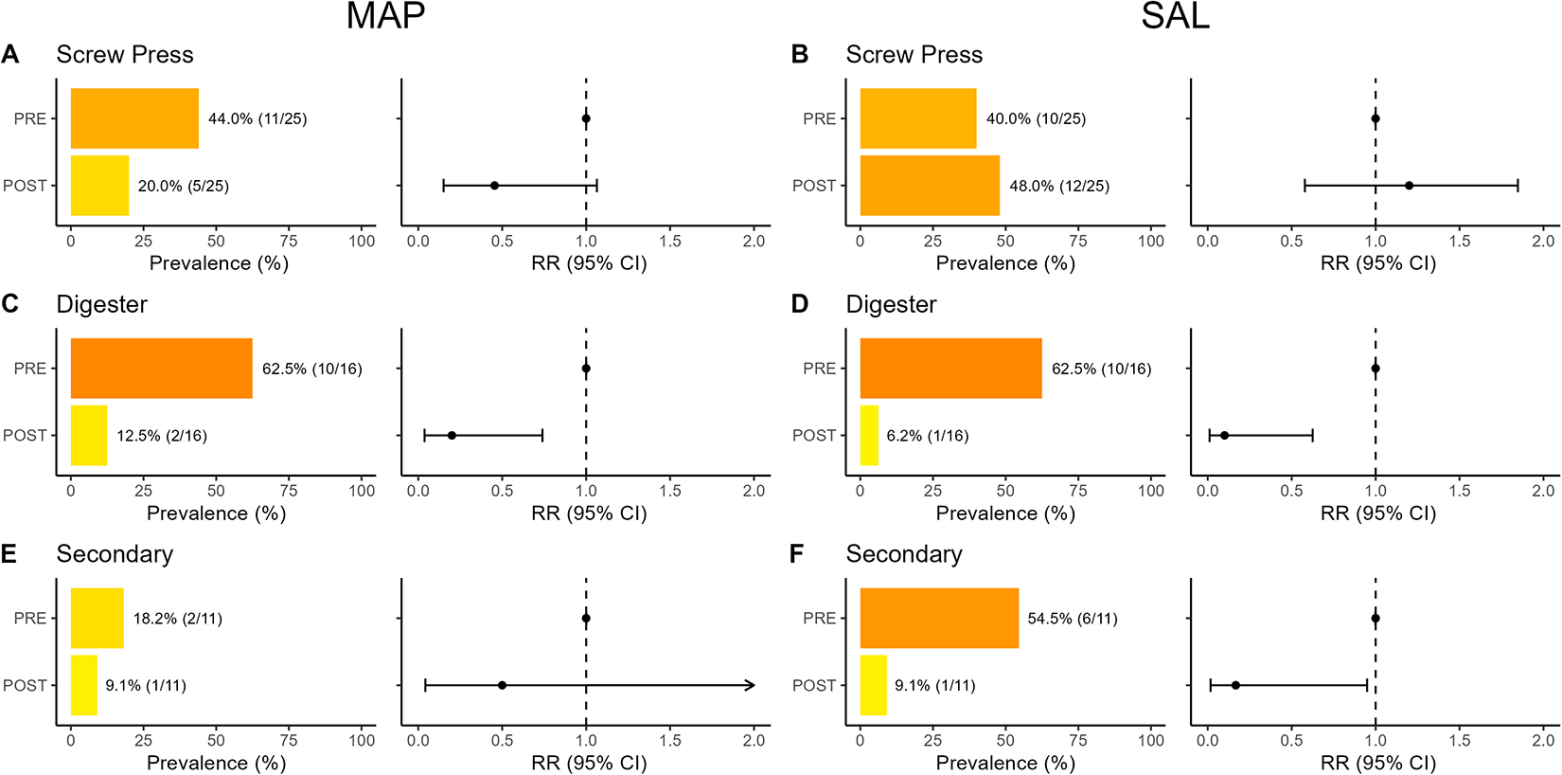
Prevalence of *Mycobacterium avium* subspecies *paratuberculosis* (MAP; panels A, C, E) and *Salmonella* (SAL; panels B, D, F) before (PRE) and after (POST) each processing step within the processing system (screw press [n = 25], digester [n = 16], or secondary processing step [DRY, COM, IR; n = 11]). Relative risks (RR) with 95% CI were estimated using logistic regression. Bars are color-coded from yellow to dark orange, with a shift toward dark orange indicating a higher prevalence.

Taken together, our results suggest that although DIG or SEC processing reduces MAP and SAL risks in RTU RMS bedding, only their combination was associated with no detectable MAP and SAL. Although our findings should be interpreted with caution due to our small sample size, our results align with previous studies on the impact of DIG on MAP (Mazzone et al., 2018; Chiapetta et al., 2019; Donat et al., 2019). A previous farm-level study showed that mesophilic DIG reduced SAL prevalence (Burch et al., 2018), whereas another study found no reduction during mesophilic DIG but elimination after COM and attributed this to higher temperatures (Chiapetta et al., 2019). Our results also align with greater SAL elimination in thermophilic versus mesophilic DIG (Qi et al., 2018). Overall, the results suggest that mesophilic DIG alone does not lead to the absence of detectable MAP and SAL. Therefore, combining DIG with SEC processing methods may be necessary to reduce the risks associated with the use of RMS bedding.

Our study is the first to investigate the presence of mastitis pathogens, MAP and SAL, in Midwest commercial dairy farms using GRN, DIG, SEC, or combined DIG+SEC RMS processing systems. Because used bedding (i.e., from the stalls) can be influenced by various management factors unrelated to the processing system itself (Peña Mosca et al., 2024), we focused our analysis on RTU solids. We attempted to minimize confounding by assessing samples collected both before and after each processing step within each herd. However, as an observational study, residual confounding may still be present, and results should be interpreted with caution, particularly when comparing the presence or counts of pathogens across farms. Limitations include a small sample size, particularly for COM and IR and the fact that the study was conducted only during summer months, which may influence study outcomes (Burch et al., 2018). This is especially critical for the comparison of the presence of MAP and SAL before versus after each step. Specifically, only relatively large differences of ∼37.4%, 47.0%, and 56.2% could have been detected in the evaluation of the screw press, DIG, and SEC, respectively, with 80% power, an α of 0.05, and assuming an initial prevalence of 80%. Similarly, for mastitis pathogen counts, only relatively large mean differences ranging from ∼1.0 to 3.0 log_10_ cfu/cm^3^ could have been detected given the available sample sizes (∼8 farms per group), the observed variability, and the same assumptions (power: 0.80 and α: 0.05). This highlights that our study might not have been able to capture more subtle variations in the presence or counts of mastitis and nonmastitis pathogens related to the processing system used. Previous studies have also shown that pooled fecal or environmental samples vary in their ability to detect herd-level presence of MAP (Wells et al., 2003; Lombard et al., 2006) and SAL (Lombard et al., 2012), with reported sensitivities ranging from 63% to 76% for MAP and 85% for SAL detection. Given the imperfect sensitivity of the methods used, the high prevalence of these pathogens, and their influence on negative predictive values, caution is needed when claiming the complete absence of pathogens. Our sampling approach (collecting material from the surface of RTU bedding piles) is consistent with previous studies conducted by our group (Patel et al., 2019; Godden et al., 2023; Peña Mosca et al., 2024). However, it is important to acknowledge that this method may not fully represent the microbial populations present deeper within the unused RMS bedding pile, which remains to be investigated. Finally, further research is needed to assess the relative economics and return on investment for adopting these individual or combined processing systems.

Compared with GRN, DIG-only or SEC-only processing methods were associated with a numerical or statistical decrease in the level of mastitis pathogens and decreased risk for the presence of SAL and MAP in RTU RMS bedding, though these pathogens were still identified. However, the combined use of these systems (DIG+SEC) was associated with the greatest reduction in the counts of mastitis pathogens and the nondetection of MAP and SAL in RTU bedding. We recommend that further research be conducted to verify the repeatability of these findings, focusing on additional combinations of processing systems (e.g., DIG+COM, DIG+DRY or DIG+IR), increasing the number of dairies evaluated for each processing method, and sampling dairies across multiple seasons.
